# Dimensionality Controls Anion Intermixing in Electroluminescent Perovskite Heterojunctions

**DOI:** 10.1021/acsphotonics.2c00604

**Published:** 2022-07-07

**Authors:** Sang-Hyun Chin, Lorenzo Mardegan, Francisco Palazon, Michele Sessolo, Henk J. Bolink

**Affiliations:** Instituto de Ciencia Molecular (ICMol), Universidad de Valencia, C/Catedrático J. Beltrán 2, 46980 Paterna, Spain

**Keywords:** perovskites, heterojunctions, light-emitting diodes, light-emitting electrochemical cells, vacuum deposition, ion-diffusion

## Abstract

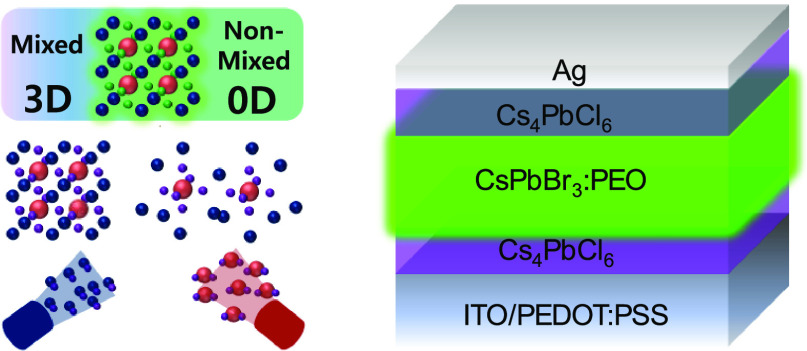

Metal halide perovskites have emerged as a promising group of materials for optoelectronic applications such as photovoltaics, light emission, and photodetectors. So-far, in particular, the stability of light-emitting devices is limited, which is in part attributed to the intrinsic ionic conductivity of these materials. High-performance devices inevitably contain heterojunctions similar to other optoelectronic devices based on oxide perovskites, II–VI, or III–V group semiconductors. To enable efficient heterojunctions, ion exchange at the interface between different layers should be controlled. Herein, we report a method that enables to control and monitor the extent of anion intermixing between solution-processed lead bromide and vacuum-deposited lead chloride perovskite films. Taking advantage of the ability to fine tune the layer thicknesses of the vacuum-deposited films, we systematically study the effect of film thickness on anionic intermixing. Using these multiple layers, we prepare proof of principle light-emitting devices exhibiting green and blue electroluminescence.

## Introduction

In modern optoelectronics, epitaxial heterostructures have been employed to achieve superior device performances.^1−3^ However, in the case of metal halide perovskites, there are only a few reports of perovskite/perovskite heterojunctions published to date.^4−6^ This is mainly due to processing limitation, especially when different materials are coated from solutions of similar polarity, and to the tendency of halide perovskites to exchange anions.^7,8^ Developing perovskite–perovskite heterojunctions could therefore improve performances and diversify advanced applications. Besides three-dimensional (3D) cesium lead trihalide perovskites (CsPbX_3_), several lower dimensional cesium lead halide analogues, such as 2D CsPb_2_X_5_ and 0D Cs_4_PbX_6_ (where X = Cl^–^, Br^–^, I^–^), exist.^9,10^ Thin films of these materials can be deposited by adjusting the deposition rates of the precursors during vacuum co-evaporation.^11,12^ Cs_4_PbX_6_ typically shows a high exciton binding energy and a low electronic conductivity, the properties of which can be useful in light-emitting applications. For instance, Liashenko *et al.* blended Cs_4_PbBr_*x*_Cl_6–*x*_/CsPbBr_3_Cl_3–*x*_ in polyethylene oxide (PEO), which was deposited between indium tin oxide (ITO) and indium gallium (InGa) eutectic electrodes, to fabricate single layer light-emitting diodes (LEDs).^13^ The Cs_4_PbBr_*x*_Cl_6–*x*_/CsPbBr_3_Cl_3–*x*_ blend showed high photoluminescence quantum yield (PLQY) and good spectral stability thanks to the suppression of halide migration. The latter effect is a consequence of the reduced ionic mobility in the 0D Cs_4_PbBr_*x*_Cl_6–*x*_ phase, as reported elsewhere.^12,13^

Apart from the control over the material stoichiometry and thickness, vacuum co-evaporation allows the fabrication of multi-layer architecture.^14^ In this work, we study perovskite heterojunctions combining 3D CsPbBr_3_ with 0D Cs_4_PbCl_6_. CsPbBr_3_ is employed in a blend with PEO, whose electron lone pairs interact with lead halides resulting in higher PLQYs.^15,16^ In addition, this polymer–perovskite mixture can form compact films without anti-solvent treatment.^17,18^ In this work, we deposit perovskite heterojunctions and investigate ion diffusion as well as their optoelectronic properties, in particular by incorporating the heterostructures in LEDs. With this simple structure, it is possible to exclude the effect of transport layers and isolate the physical properties of the perovskite heterojunction.

## Results and Discussion

**Figure 1:**
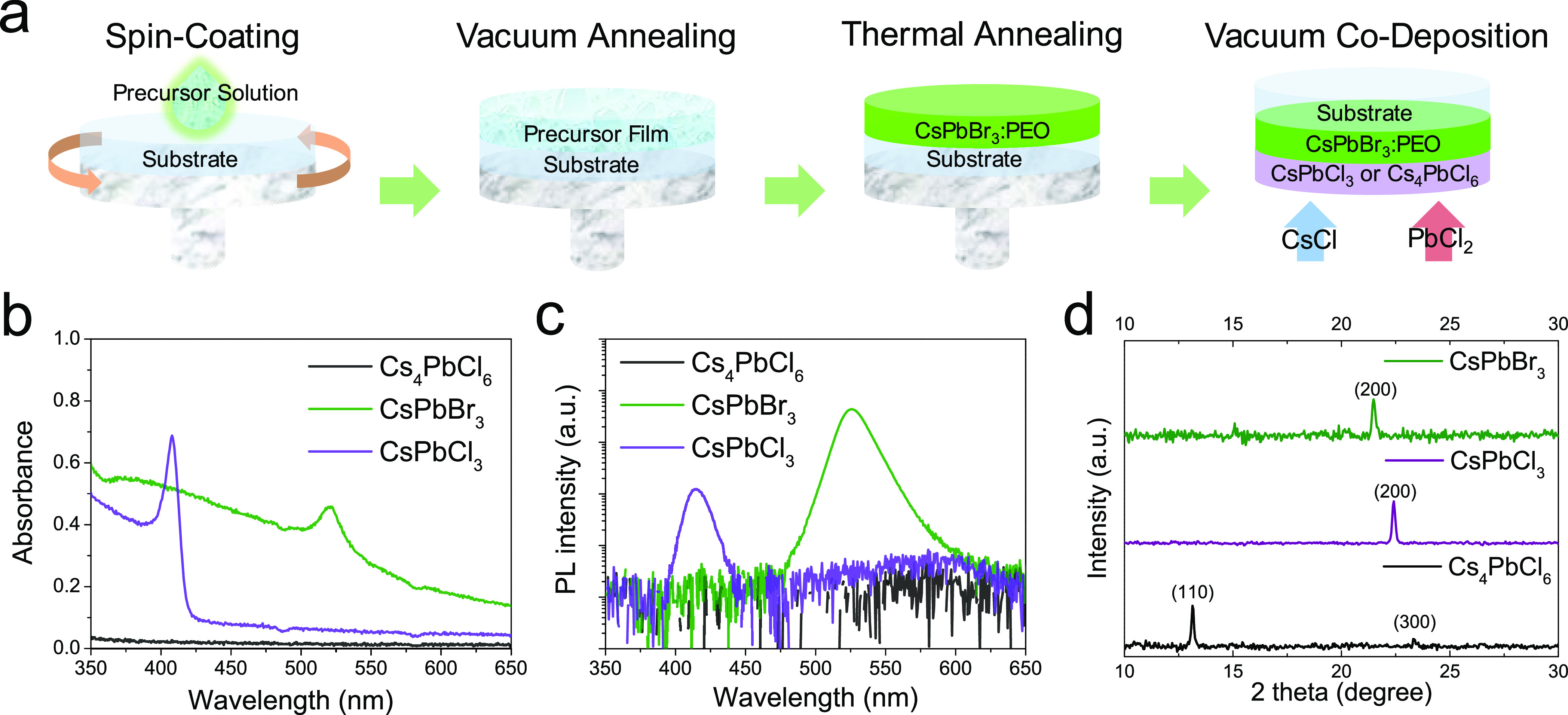
(a) Schematic illustration of the fabrication of lead halide perovskite heterojunctions. (b) Absorbance and (c) photoluminescence spectra (excitation wavelength: 375 nm) and (d) X-ray diffraction patterns of pristine CsPbBr_3_, CsPbCl_3_, and Cs_4_PbCl_6_ films.

The general deposition method is illustrated in Figure 1a. CsPbBr_3_ (225 mg, CsBr:PbBr_2_ molar ratio is 1.5:1) is blended with PEO (150 mg, molecular weight ≈ 600,000 u) and dissolved in 8 mL of dimethyl sulfoxide (DMSO). The solution is then spin-coated on the ITO/PEDOT:PSS layer followed by annealing in vacuum to promote nucleation and formation of compact films.^18,19^ After this process, the samples are thermally annealed at 70 °C for 3 min to eliminate the residual solvent. The chloride-based films are deposited by vacuum co-sublimation of cesium chloride and lead chloride on top of the solution-processed CsPbBr_3_:PEO films. The dimensionality of the cesium lead chloride compounds is controlled via the relative deposition rates of the two precursors. Unlike in previous reports, in this study, we do not add any salts to improve the overall device performances.

The optical absorption spectra for our solution-processed CsPbBr_3_ and vacuum-deposited CsPbCl_3_ and Cs_4_PbCl_6_ films are presented in Figure 1b. CsPbBr_3_ and CsPbCl_3_ show the characteristic excitonic peaks at 520 and 400 nm, respectively.^20^ Cs_4_PbCl_6_ does not show any absorption in the entire visible region, in view of its band gap of 4.37 eV.^21^ Upon excitation with a 375 nm laser, CsPbBr_3_ and CsPbCl_3_ show photoluminescence (PL) peaks centered at 528 and 405 nm, respectively (Figure 1c), with a small Stokes shift characteristic of direct-band gap bulk 3D perovskites.^20^ In the case of Cs_4_PbCl_6_, we did not observe any PL signal because its band gap is larger than the photon energy at 375 nm. Figure 1d shows the XRD patterns for these three films. The diffractograms of the 3D perovskites show a main peak at 2θ = 21.5° for CsPbBr_3_ and 2θ = 22.5° for CsPbCl_3_, which can be ascribed to the (200) plane, which is expected to be the main diffraction signal for non-oriented crystals (see Inorganic Crystal Structure Database, ICSD, references 243734 and 243735). Two characteristic reflections for the 0D Cs_4_PbCl_6_ are observed at 2θ = 13.1 and 2θ = 23.3° (see Inorganic Crystal Structure Database, ICSD, references 35703). Note that there is no diffraction peak around a 2θ of 22.5° for the XRD pattern of Cs_4_PbCl_6_, which is typical of the (200) plane of the CsPbCl_3_, which will be helpful in the characterization of the corresponding bilayers (see next section).

**Figure 2:**
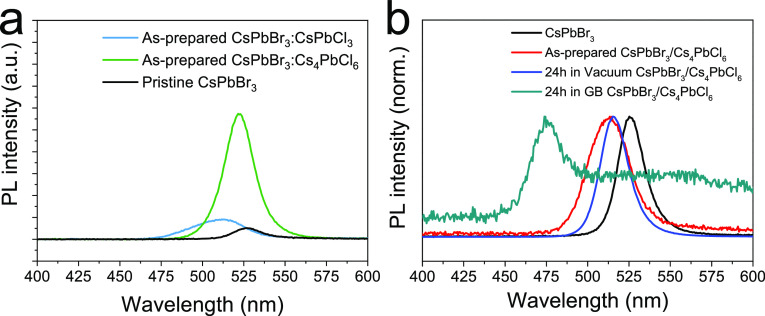
(a) Photoluminescence spectra of as-prepared CsPbBr_3_, CsPbBr_3_/CsPbCl_3_, and CsPbBr_3_/Cs_4_PbCl_6_ films, and (b) spectra of CsPbBr_3_ and CsPbBr_3_/Cs_4_PbCl_6_ stored in a vacuum chamber and N_2_ glovebox (excitation wavelength: 375 nm).

With these three films, heterojunction stacks are fabricated by combining spin-coating and vacuum co-evaporation to investigate the ion-diffusion property at the interface of these materials.^4^ We initially studied the PL of a 3D/3D heterojunction of the type CsPbBr_3_ (300 nm)/CsPbCl_3_ (50 nm) using a 375 nm laser excitation source (Figure 2a). The as-prepared CsPbBr_3_/CsPbCl_3_ bilayers show a blue-shifted and asymmetric PL signal with respect to that of the pure CsPbBr_3_, corresponding to a cyan color emission. This observation is indicative of spontaneous anion exchange between the two materials, even at room temperature. In contrast, the 3D/0D CsPbBr_3_/Cs_4_PbCl_6_ heterojunction shows a negligible PL shift and also a more intense PL as compared to the reference CsPbBr_3_. This observation is in good agreement with the previous report by Shen *et al.* who showed hindered anion diffusion and mixing in 0D Cs_4_PbX_6_ (X = Cl^–^, Br^–^).^22^

The PL of 3D/0D CsPbBr_3_/Cs_4_PbCl_6_ bilayers was further studied as a function of the storage condition (Figure 2b). We observed a blue shift of the PL signal after storing the bilayer for 24 h in a N_2_ atmosphere. It is worth to note that this glovebox for storage experiment is a different one from that for the spin-coating, hence the atmosphere is also free of any solvent vapor.

On the contrary, when stored in a vacuum evaporator (base pressure: 2·10^–6^ mbar) for the same time, the CsPbBr_3_/Cs_4_PbCl_6_ maintained the initial PL peak position. Also, the PL peak was found to be sharper after storing the material in vacuum. This interesting observation agrees with a previous report by Karlsson *et al.*, who showed that in vacuum the material can reorganize and achieve a superior compositional homogeneity, in turn leading to a narrower PL peak.^23^

**Figure 3:**
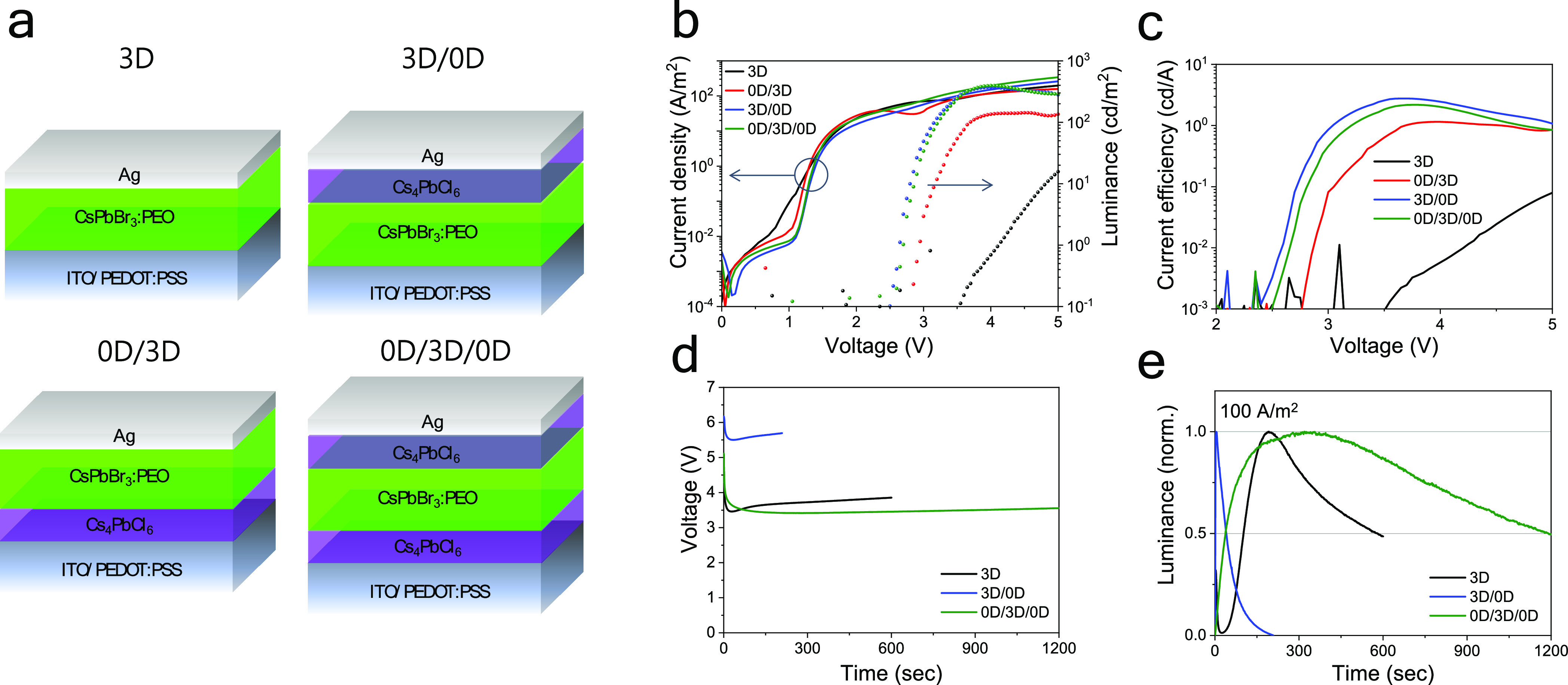
(a) Schematic illustration of the perovskite heterojunction-based light emitters. (b) Current density (lines) and luminance (symbols) vs applied voltage for the same devices. The corresponding current efficiency is shown in (c). (d) Time-dependent voltage and (e) luminance for the same devices driven with a constant current density of 100 A/m^2^.

In view of the absence of halide mixing in the 3D/0D heterojunctions (CsPbBr_3_/Cs_4_PbCl_6_), as observed by PL, we prepared planar devices combining 0D and 3D metal halide films, in order to study their electroluminescent behavior. In particular, we used CsPbBr_3_:PEO as the light emitters and studied the effect of the 0D Cs_4_PbCl_6_ layer below (at the anode, 0D/3D), on top (at the cathode, 3D/0D), and on both sides of the 3D CsPbBr_3_ emitter (0D/3D/0D triple layers). The structure of the devices is illustrated in Figure 3a, and they are named following the order and dimensionality of the corresponding heterojunctions. In addition, the flat band diagram is described in the Supporting Information, Figure S1. Since Cs_4_PbCl_6_ is a zero-dimensional material with chloride, it forms a type-I structured heterojunction with CsPbBr_3_ perovskite.

The current density and luminance vs voltage (JVL) curves for this device set is reported in Figure 3b. The heterojunction-based devices show a slightly reduced leakage current below 1 V, as compared to the reference single-layer 3D light emitter. More importantly, all heterojunction devices exhibited lower turn-on voltage and more intense electroluminescence as compared to the reference 3D device. In the perovskite heterojunctions, 25 nm thick Cs_4_PbCl_6_ films are deposited. After deposition, the main PL peak is slightly blue shifted, suggesting halide intermixing at the 3D/0D interface. Hence, the actual Cs_4_PbCl_6_ layer is even thinner compared to the nominal deposited thickness. In this scenario, it is plausible that the lower turn-on voltage originates from interfacial band bending effects favored by the thin Cs_4_PbCl_6_ films.

The 3D/0D and 0D/3D/0D devices reached luminance level of about 400 cd/m^2^, at applied bias of approximately 3.5 V. At the same voltage, device 0D/3D showed lower EL intensity, about one third as compared to the others. As the current density (both in magnitude and profile) is very similar for these three types of devices, their current efficiency (Figure 3c) follows the trend in luminance. The reference 3D device shows a low current efficiency with a maximum of 0.1 cd/A at 5 V. With the 0D layer at the anode (device 0D/3D), the efficiency maximum was improved to 1 cd/A at 4 V, which is further improved when the Cs_4_PbCl_6_ 0D film is placed in between the CsPbBr_3_ 3D emitter and the cathode. Device 3D/0D and device 0D/3D/0D showed superior efficiency (>3 cd/A), indicating that non-radiative recombination at the CsPbBr_3_/Ag interface is strongly reduced upon insertion of the 0D buffer layer (as the current density is unvaried between the different device configuration).

In addition to the enhanced performance, we observed a significant improvement in the operational stability of the devices 3D/0D and 0D/3D/0D (Figure 3d,e). To test the stability, the devices were driven with a constant current density of 100 A/m^2^, while monitoring the evolution of the luminance and voltage. Device 3D/0D was found to be unstable (only a few seconds of operation), while the pristine CsPbBr_3_-based device shows a half lifetime (*t*_1/2_, time to reach half of the maximum luminance) of approximately 10 min. Interestingly, the triple-layer heterojunction device 0D/3D/0D demonstrated a further improved operational stability: with *t*_1/2_ = 20 min. The time to reach maximum luminance, 100 cd/m^2^, in this 0D/3D/0D device was only 25 s (Supporting Information, Figure S2). In addition, this device shows negligible voltage variation (<0.1 V), indicating stable electrical properties, which might arise from the limited and balanced ion movement of the Cs_4_PbCl_6_ buffer layers. To clarify the first voltage applied, the initial time vs voltage graph in constant current operation is shown in the Supporting Information, Figure S3. The pristine device and device 0D/3D/0D only need to go through ∼4 V while device 3D/0D needs ∼6 V to apply 100 A/m^2^. The mechanism responsible for the enhanced stability of the 0D/3D/0D heterojunctions is difficult to identify precisely. It is possible that unbalanced charge injection and subsequent charge accumulation in bilayers might favor non-radiative recombination and trigger the degradation of the perovskite films. Correspondingly, the operational lifetime will be decreased significantly.^24,25^

Most of all, this limitation on the ion movement by Cs_4_PbCl_6_ makes stable electroluminescence. Even after 20 min of the device operation with 100 A/m^2^, device 0D/3D/0D does not show a blue shift, which stems from halide intermixing. On the contrary, there is a small red shift happening at about 0.2 nm/min (Figure S4) and a recovery of the green emission from CsPbBr_3_ (from 518 nm to 522 nm). The absorbance, PL, and EL spectra are reported in Figure S5. It is possible that EL recovery in this 0D/3D/0D device originates from the Cl anion diffusing out the radiative recombination zone. After 20 min of device operation, the EL peak (522 nm) becomes similar to that of the pristine device, 526 nm. This phenomenon supports our point that the heterojunction based on 0D cesium lead halide has less chance to donate a halide anion, which causes an emission peak shift.

**Figure 4:**
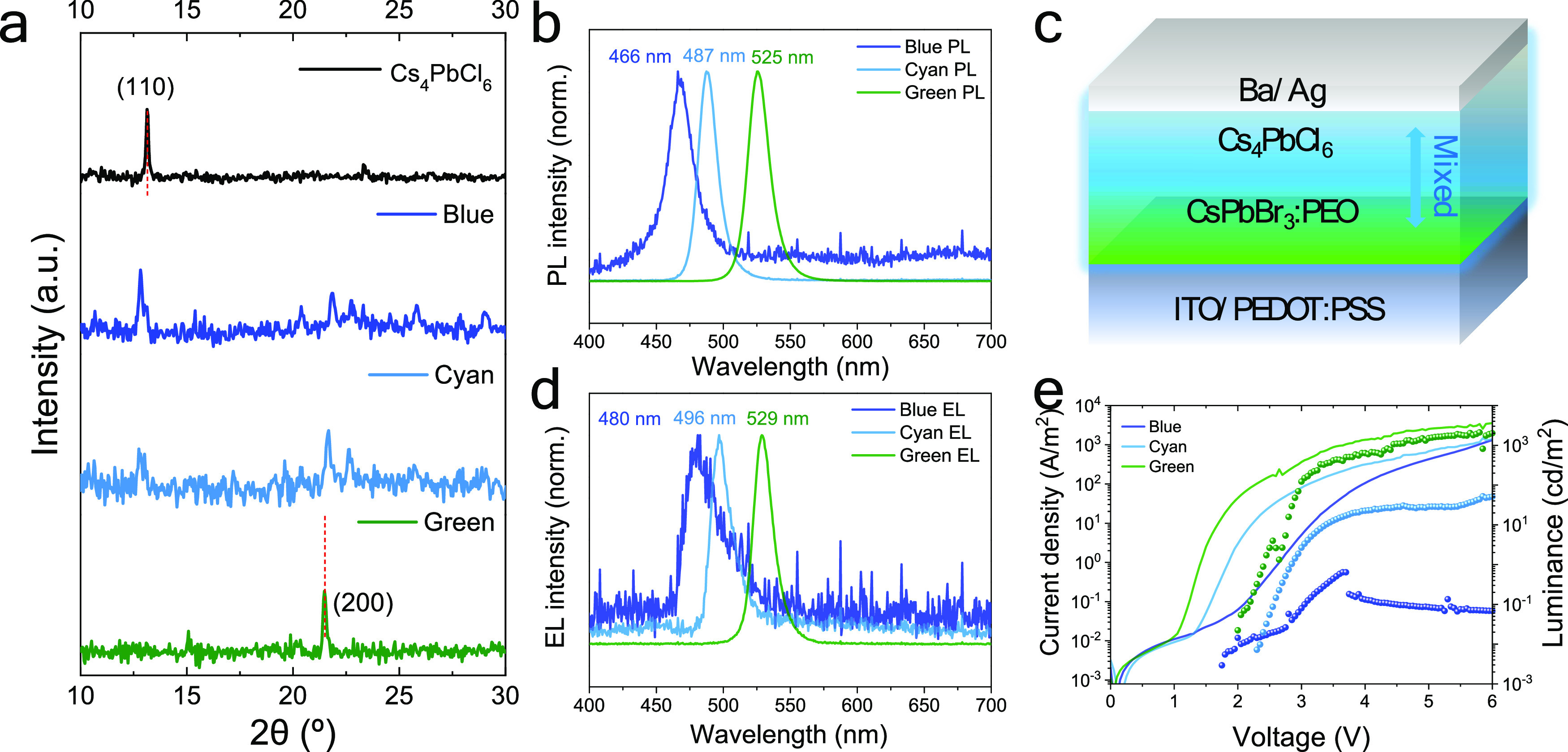
(a) XRD patterns of CsPbBr_3_, Cs_4_PbCl_6_, and mixed-halide films obtained by diffusion between them. (b) PL spectra of green-emissive CsPbBr_3_, mixed cyan, and blue-emitting films. (c) Schematic illustration and (d) EL and (e) *J*–*V*–*L* data of the light emitters based on the pristine and mixed perovskites.

While avoiding ion diffusion and mixing can be exploited to tune the optoelectronic characteristic of devices (as shown above), controlled anion-mixing is of interest to tune the emission color of perovskite films. Hence, we prepared a series of CsPbBr_3_/Cs_4_PbCl_6_ 3D/0D bilayers with constant CsPbBr_3_ thickness (300 nm) and with different Cs_4_PbCl_6_ top-layer thicknesses (0, 25, and 50 nm). These bilayers were treated via solvent vapor annealing (SVA, details in the experimental part) using a modified condition from a previous report, as SVA induces a higher PL intensity compared to slow and spontaneous mixing in nitrogen (Supporting Information, Figure S6).^26^ From the XRD patterns (Figure 4a), the main (200) diffraction peak, characteristic of the orthorhombic CsPbBr_3_ phase, shifts to higher angles after anion intermixing with the top layers, although the diffraction intensity is strongly reduced. This is due to partial bromide substitution with chloride, resulting in a smaller unit cell for mixed-halide CsPbBr_3–*x*_Cl_*x*_). Importantly, the (110) reflection at 2θ = 12.7°, corresponding to the 0D Cs_4_PbCl_6_ phase, is observed even after SVA treatment. This implies that the 3D/0D heterojunction is structurally preserved in spite of halide mixing.

The PL spectra of the CsPbBr_3_, CsPbBr_3_/Cs_4_PbCl_6_ (25 nm), and CsPbBr_3_/Cs_4_PbCl_6_ (50 nm) films show sharp and symmetric peaks centered at 525 (green), 487 (cyan), and 466 nm (blue), respectively (Figure 4b). Thus, by controlling anion intermixing through the material dimensionality (3D/0D) and the thickness of the 0D film, it is possible to fine tune the emission of the perovskite heterojunction. Interestingly, the PL quantum yield (PLQY) of the three materials do not follow a monotonic trend, with the cyan emitter having more efficient PL (14.0%) as compared to the green (9.5%) and blue (3.6%) materials. The same set of materials were incorporated in thin-film LEDs with the structure depicted in Figure 4c, where the perovskites are sandwiched between an ITO/PEDOT:PSS anode and a Ba/Ag cathode. The Ba layer is employed to ensure ohmic electron injection, similar to previous reports where LiF interlayers were used.^18,19^ We note that the blue emitter is not operational without the Ba layer. As shown in Figure 4d, the EL spectra for the three materials are only slightly red-shifted as compared to their corresponding PL. The JVL curves for three representative devices are reported in Figure 4e. The maximum measured luminance values were 2000 and 100 cd/m^2^ for green and cyan emitters, respectively, which is comparable to other recent reports on similar CsPbX_3_:PEO perovskite emitters.^13,18,19^ We observed a low current density and luminance for the blue emitter, which might be related with the thick insulating Cs_4_PbX_6_ 0D top layer. In general, the current efficiency during *J*–*V* scan was found to be relatively low (<1 cd/A, Supporting Information, Figure S7).

## Conclusions

In summary, perovskite heterojunctions were successfully fabricated by subsequent solution-process and vacuum co-evaporation. The halide mixing properties of 3D/3D and 3D/0D bilayers were evaluated, and we found that the CsPbBr_3_/Cs_4_PbCl_6_ heterojunction showed limited anion intermixing, leading to a structurally stable 3D/0D interface. These heterostructures can be exploited in light-emitters, where the triple-layer 0D/3D/0D heterojunction was found to be more efficient and stable as compared to the 3D/0D or 0D/3D bilayers. Also, we achieved fast response, electron transport layer-free CsPbBr_3_ light emitters with reasonable luminance compared to recent reports, without adding any ionic salt. This work proposes a way to stabilize or even suppress anion intermixing at the perovskite heterojunction, which is relevant for the future development of perovskite LEDs. Additionally, we also showed that in these bilayers, it is possible to promote halide mixing when the bilayers are exposed to solvent vapor. Using this method, the photo- and electroluminescence can be tuned from green to blue by adjusting the thickness of the 0D Cs_4_PbCl_6_ top layer.

## Experimental Section

### Materials

Cesium bromide (CsBr, 99.9%) is purchased from Alfa Aesar, and lead bromide (PbBr_2_, 98%), cesium chloride (CsCl, 99.999%), polyethylene oxide (PEO, Mw ≈ 600,000), and dimethylsulfoxide (DMSO, anhydrous) are purchased from Sigma Aldrich. Lead chloride (99.999%) is purchased from Lumtec. Poly(3,4-ethylenedioxythiophene) polystyrene sulfonate (PEDOT:PSS, AI 4083) is purchased from Clevios.

### Preparation

Indium tin oxide (ITO) substrate (3 cm × 3 cm) is cleaned with detergent, tap water, deionized water, and isopropanol for 5 min, and this substrate is treated in UV ozone for 20 min before the spin-coating of PEDOT:PSS (4000 rpm, 30 s). The PEDOT:PSS film is thermally treated with 150 °C 10 min. This film should be cooled-down before the perovskite deposition.

A total of 105 mg of CsBr, 120 mg of PbBr_2_, and 150 mg of PEO are mixed in 8 mL of DMSO and stirred at 50 °C overnight. The solution was cooled down before spin-coating (1000 rpm 60 s in a N_2_ glovebox) on the PEDOT:PSS film. Right after spin-coating, the sample is transferred into the antechamber of the glovebox and vacuum-treated for 90 s. Finally, the perovskite film is annealed at 70 °C on a hot plate for 3 min.

For the subsequent deposition of the cesium lead chloride films, the CsPbBr_3_ perovskite film is transferred into a second glove box with an integrated vacuum chamber. The precursors are thermally evaporated in a chamber (custom made by Thermal Vacuum Projects) with a base pressure of 10^–6^ mbar. The deposition rate for PbCl_2_ is kept constant at 0.5 Ȧ/s, while the deposition rates used for CsCl were 1.7 and 0.4 Ȧ/s for Cs_4_PbCl_6_ and CsPbCl_3_, respectively. A calibration factor was obtained by comparing the thickness inferred from the quartz crystal microbalance (QCM) sensors with that measured with a mechanical profilometer (Ambios XP1).

### Characterization

Absorption spectra were collected using a fiber optics-based Avantes, Avaspec2048 spectrometer. The photoluminescence spectra were also measured with an Avantes, Avaspec2048 spectrometer, and films were illuminated with a diode laser of integrated optics, emitting at 375 nm. The crystalline structure of the film samples was studied by XRD. The patterns were collected in Bragg–Brentano geometry on an Empyrean PANalytical powder diffractometer with a copper anode operated at 45 kV and 40 mA. Further analysis, including Le Bail fits, was performed with Fullprof software. The devices were measured by applying a constant current density of 100 A/m^2^ while monitoring the voltage and luminance versus time.

## Supporting Information Available

Band diagrams, supplementary electrical characterization, complementary optical absorption, photoluminescence, electroluminescence (PDF)
