# Supplementary material for: Dimensionality Controls Anion Intermixing in Electroluminescent Perovskite Heterojunctions
Source: ACS Photonics. 2022 Jul 7;9(7):2483–8. doi: 10.1021/acsphotonics.2c00604 (PMC9305999; doi:10.1021/acsphotonics.2c00604)
Supplement: Supplementary file 1 — ph2c00604_si_001.pdf [file ph2c00604_si_001.pdf]

Dimensionality Controls Anion Intermixing in Electroluminescent Perovskite Heterojunctions

Sang-Hyun Chin, Lorenzo Mardegan, Francisco Palazon, Michele Sessolo, and Henk J. Bolink**

Instituto de Ciencia Molecular (ICMol), Universidad de Valencia, C/ Catedrático J. Beltrán 2, 46980 Paterna, Spain

AUTHOR INFORMATION

Corresponding Authors

Michele Sessolo, Henk J. Bolink - Instituto de Ciencia Molecular (ICMol), Universidad de Valencia, C/ Catedrático J. Beltrán 2, 46980 Paterna, Spain; orcid.org/0000-0001-9784-6253;

E-mail: michele.sessolo@uv.es; henk.bolink@uv.es.

Keywords: perovskites, heterojunctions, light-emitting diodes, light-emitting electrochemical cells, vacuum deposition, ion-diffusion

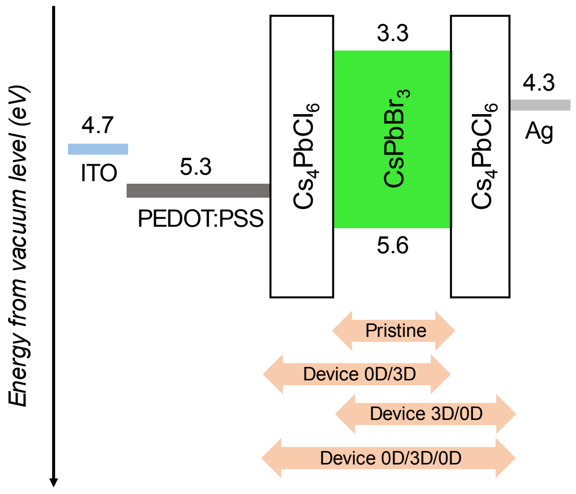

Figure S1. Schematic flat band energy diagram of the materials used in light-emitting devices based on $Cs_4PbCl_6/CsPbBr_3$ heterojunctions. Values for $CsPbBr_3$ are from the literature.[1] The reported IE for the iodide compound Cs_4PbI_6 is 7.15 eV;[2] considering a downward shift of the valence band maximum (VBM) when exchanging I for Cl (due to increased electronegativity),[3] we expect the IE of Cs_4PbCl_6 to be approximately 7.5 eV. Taking into account an optical bandgap of 4.4 eV, the electron affinity (EA) of Cs_4PbCl_6 would be of about 3 eV.

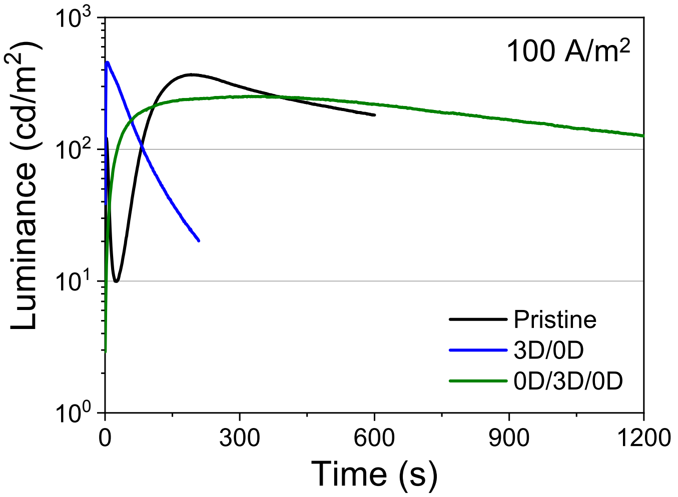

Figure S2. Constant current driving with $CsPbBr_3$ (Pristine), $CsPbBr_3/Cs_4PbCl_6$ (Device 3D/0D) $Cs_4PbCl_6/CsPbBr_3/Cs_4PbCl_6$ (Device 0D/3D/0D).

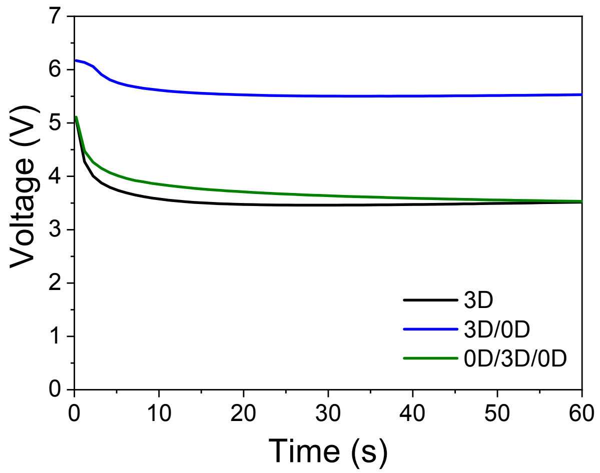

Figure S3. Zoom of the initial voltage decay upon application of a constant current of 100 A/m^2 to the light-emitting devices based on $Cs_4PbCl_6/CsPbBr_3$ heterojunction.

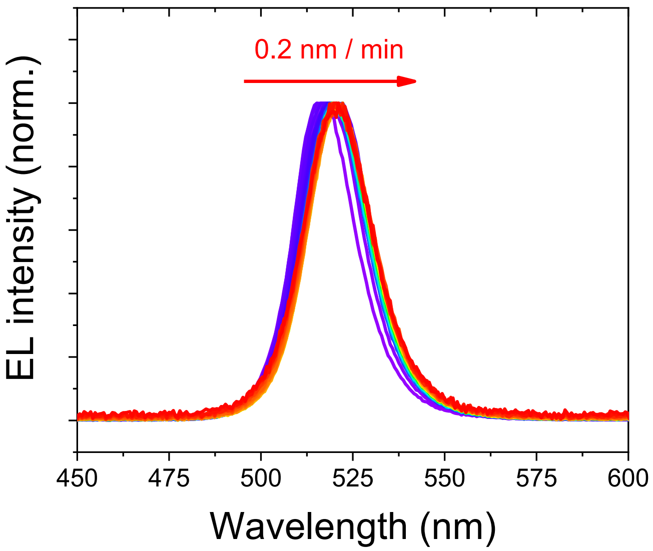

Figure S4. Electroluminescence spectra as a function of time while biasing 0D/3D/0D device ($Cs_4PbCl_6/CsPbBr_3/Cs_4PbCl_6$) at 100 A/$m^2$ for 20 minutes.

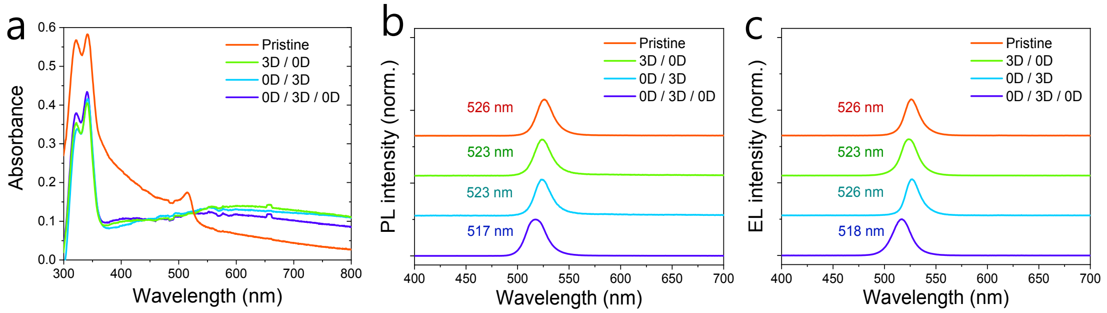

Figure S5. a) Optical absorption, b) photo- and c) electro-luminescence spectra from pristine $CsPbBr_3$:PEO (3D) films, 3D/0D, 0D/3D and 0D/3D/0D bi- and tri-layer perovskite heterojunctions.

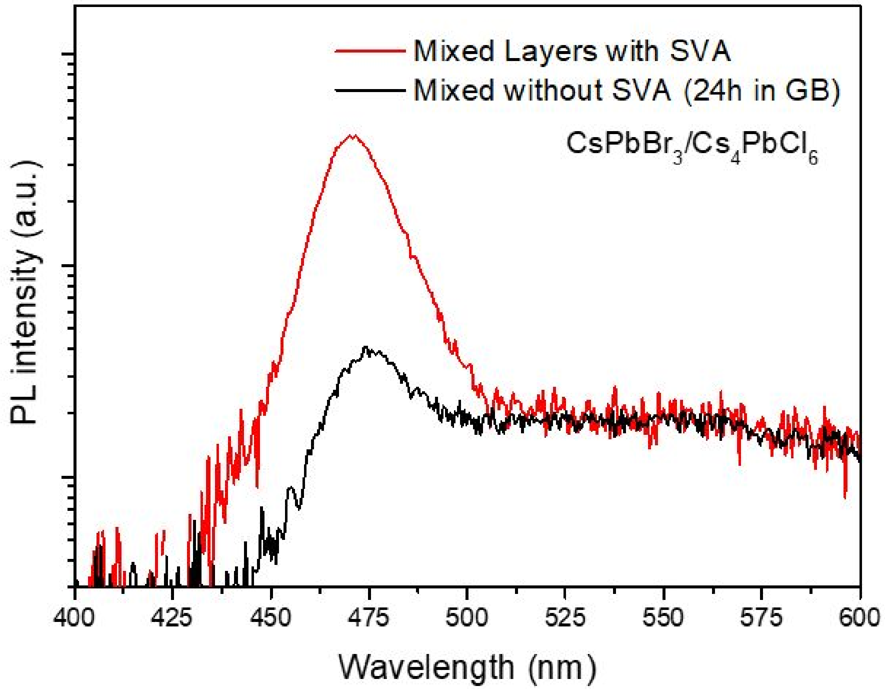

Figure S6. Photoluminescent spectra of intermixed $CsPbBr_3/Cs_4PbCl_6$ with and without solvent vapor annealing (SVA).

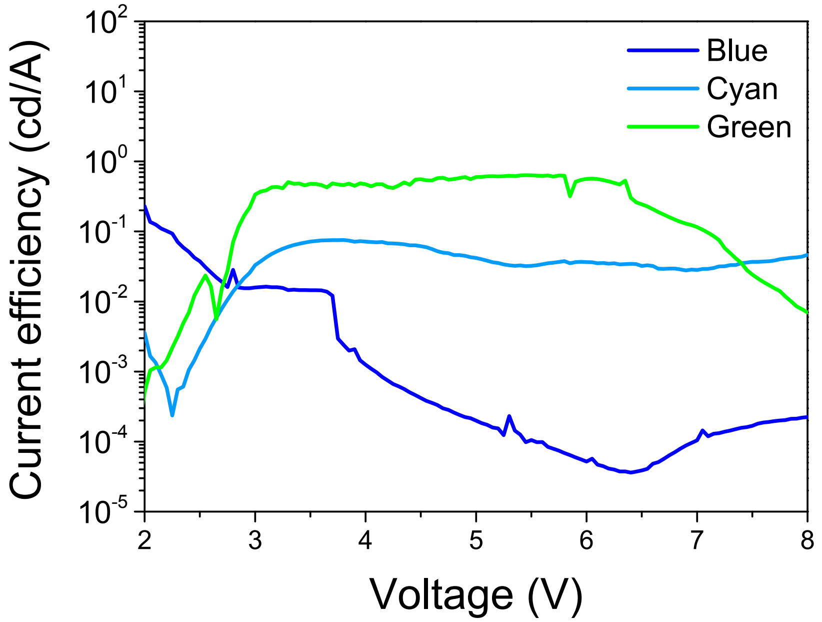

Figure S7. Current efficiency of $CsPbBr_3$ and Mixed $CsPbBr_3/Cs_4PbCl_6$-based devices.

References

(1) J. Endres, M. Kulbak, L. Zhao, B. P. Rand, D. Cahen, G. Hodes, A. Kahn, *J. Appl. Phys.* **2017**, *121*, 035304.

(2) J. Yin, J.-L. Brédas, O. M. Bakr, O. F. Mohammed, *Chem. Mater.* **2020**, *32*, 5036.

(3) S. Tao, I. Schmidt, G. Brocks, J. Jiang, I. Tranca, K. Meerholz, S. Olthof, *Nat. Commun.* **2019**, *10*, 2560.
